# Dexmedetomidine for LISA procedure: a prospective observational single center experience

**DOI:** 10.1186/s13052-026-02202-z

**Published:** 2026-02-04

**Authors:** Beatrice Galeazzo, Francesca Tormena, Cinzia Anna Maria Papappicco, Serena Gomirato, Silvia Vendramin, Dario Gregori, Paola Lago

**Affiliations:** 1 Department of Critical Care, Neonatal Intensive Care Unit, Azienda ULSS2-Marca Trevigiana, Ca’ Foncello Hospital, Treviso, Italy; 2 Unit of Biostatistics, Epidemiology and Public Health-University of Padua, Padua, Italy

**Keywords:** RDS, LISA, Sedation, Dexmedetomidine

## Abstract

**Background:**

Respiratory Distress Syndrome (RDS) is the most common respiratory problem of preterm newborns. The preferred way to manage RDS is with Less Invasive Surfactant Administration (LISA), which involves direct laryngoscopy in spontaneously breathing infants. Despite its widespread diffusion, the optimal sedation protocol for LISA remains unclear. Dexmedetomidine, an α2-adrenergic agonist, offers sedative and analgesic properties with minimal respiratory depression. This pilot study aims to evaluate dexmedetomidine for LISA procedure focusing on the frequency of adequate sedation (defined as N-PASS score −2 to −5) and analgesia (NIPS score < 4) and adverse events related to dexmedetomidine.

**Methods:**

This is a prospective observational study conducted in our tertiary Italian NICU from May 2021 to July 2024. We enrolled preterm neonates aged between 26^+0^ to 36^+6^ weeks, diagnosed with RDS who required LISA. We also analyzed the study population into two groups based on a cut-off of 32 weeks to understand possible differences in adverse events and sedation and analgesia scores, even if this was not our prior aim. Dexmedetomidine (1 µg/kg) was administered intravenously before LISA. Primary outcomes included pain control assessed by Neonatal Infant Pain Scale (NIPS) score, sedation adequacy assessed by Neonatal Pain, Agitation, and Sedation Scale (N-PASS), and success rate of the procedure (such as laryngoscopy conditions assessed by Goldberg score and number of attempts). We also assessed the safety of the procedure evaluating adverse events, such as intubation rates, apneas/desaturations, bradycardia, and hypotension.

**Results:**

Forty-seven preterm newborns received dexmedetomidine. The median (IQR) gestational age (weeks/days), birth weight (grams) and age (hours) at LISA were respectively 29^+6^ (28^+5^, 33^+1^) weeks, 1421 (1069–2074) g, 3 (2.5–6) hours. Pain scores indicated adequate pain control during the procedure (NIPS < 4 in 76% during laryngoscopy). Light sedation (N-PASS −2 to −5) was observed in 23% of patients before the procedure, lasting up to 60 minutes. Excessive sedation (N-PASS −6 to −10) occurred in 7% of babies (3 patients). Laryngoscopy conditions were adequate in 90% of neonates (Goldberg score < 6). Apnea/desaturation with/without bradycardia occurred in 13% of newborns. Six patients were intubated (13%) within 72 hours after the procedure. Hypotension was noted in 6.4% of patients.

**Conclusion:**

Premedication with dexmedetomidine resulted in a good success rate for pain control; however, an adequate level of sedation was not consistently achieved. While the frequency of adverse events was acceptable, a more rapid-onset sedative may be preferable for this procedure. Further randomized controlled trials are needed to establish an optimal sedation strategy for LISA in preterm newborns.

**Trial registration:**

Register: ClinicalTrials.gov, number ID NCT04820101, enregistered 03–29-2021, https://clinicaltrials.gov/study/NCT04820101?cond = RDS&term =dexmedetomidine&rank = 1#study-record-dates .

## Background

Respiratory distress syndrome (RDS) is the most common respiratory problem in preterm newborn [1] The combination of exogenous surfactant administration and non-invasive ventilation is the most effective approach to manage RDS [2–4].

Among the various techniques for surfactant delivery, the European Consensus Guidelines on RDS management recommend Less Invasive Surfactant Administration (LISA) as the preferred method for spontaneously breathing newborns on CPAP with worsening RDS (FiO₂ > 0.30 on CPAP pressure ≥6 cm H₂O) [1]. This technique reduces the need for invasive mechanical ventilation during the first 72 hours, decreases the risk of the composite outcome of death and BPD and BPD alone among survivors and appears to reduce the risk of intracranial hemorrhage [5–9].

LISA involves the use of a semirigid catheter inserted through the trachea via direct laryngoscopy to administer surfactant while the infant maintains spontaneous breathing and the vocal cords sustain auto-PEEP [10].

With the widespread use of this technique, it is essential to establish a more standardized approach to sedation and analgesia for LISA. An ideal drug for this context should have a fast onset, short duration of action, rapid offset, and provide good sedation and analgesia with minimal impact on respiratory drive.

In the last decades, dexmedetomidine has become increasingly popular in neonatal and pediatric populations. It is an α2-adrenergic agonist that acts on the brainstem by inhibiting norepinephrine release, activating the receptors in the locus coeruleus and decreasing the release of substance P in the dorsal horn of the spinal cord. This leads to its sedative, anxiolytic and analgesic effects without impacting respiratory drive. It is an attractive agent to achieve procedural sedation and analgesia in spontaneously breathing newborns (e.g., neuroimaging MRI) [11–14].

With this pilot study we aim to evaluate dexmedetomidine as premedication for LISA procedure. The primary outcomes were the rate of adequate sedation (light sedation, N-PASS score between −2 and −5) and analgesia (mild pain, NIPS score < 4), the success of LISA procedure (adequate laryngoscopy conditions, number of laryngoscopy attempts, time taken to perform LISA), meanwhile the secondary outcomes were the frequencies of adverse events related to dexmedetomidine.

## Materials and methods

### Study design and population

We conducted a prospective observational study in the tertiary NICU of Ca’ Foncello Hospital, Treviso, Italy, from May 2021 to July 2024 to assess our new protocol for the LISA procedure.

**Inclusion criteria were:** gestational age at birth between 26^+0^ to 36^+6^ weeks and Respiratory Distress Syndrome (RDS) requiring surfactant therapy (worsening RDS with FiO₂ > 0.30 on CPAP pressure ≥6 cm H₂O).

The enrolled newborns were divided in two groups (26^+0^-31^+6^ and 32^+0^-36^+6^ weeks), who were hospitalized in our NICU, diagnosed with RDS and required surfactant therapy. This cutoff allows for a meaningful comparison between very preterm infants (< 32 weeks), who are physiologically more immature and at a higher risk for adverse events, and moderate-to-late preterm infants (≥ 32 weeks). While our study was not powered to be a comparative trial, this exploratory stratification was intended to help identify potential differences in drug tolerance, efficacy, and adverse event profiles between these two distinct populations.

**Exclusion criteria were:** need for emergency intubation in the delivery room, documented major congenital malformations (including cardiopathies, suspected chromosomal abnormalities, fetal hydrops), hypercapnia (defined as arterial or capillary CO₂ tension > 65 mmHg), pneumothorax confirmed on chest imaging and hemodynamic instability defined as: abnormal or unstable blood pressure for age < tenth centile) in combination with either hypoxemia (SpO₂ < 80%) or increased oxygen demand during neonatal transition, and requiring either intubation or vasoactive pharmacological support (including inotropes or vasopressors) or requiring fluid bolus resuscitation within 1 hour before planned LISA.

## Description of the procedure

Eligible infants were stabilized in the delivery room according to the ILCOR 2020 guidelines [15]. If necessary, CPAP or non-invasive ventilation was applied and continued in Neonatal Intensive Care Unit (NICU).

In accordance with the European guidelines for the management of RDS, LISA procedure was performed early in the course of the disease when the infants showed worsening symptoms, dyspnea, and required FiO₂ > 0.30 on CPAP with a pressure of at least 6 cm H₂O [1]. Point-of-care lung ultrasound and the Silverman score were not formally utilized as primary decision-making tools.

A semirigid catheter for surfactant endotracheal instillation was inserted via the mouth in spontaneously breathing infants. Direct laryngoscopy was performed, and the probe was inserted beyond the vocal cords to the required depth. Once the probe was correctly positioned, surfactant (Poractant alfa, Curosurf, CHIESI Farmaceutici, Parma, Italy) was slowly infused over 2–3 minutes at an initial dose of 200 mg/kg. At the end of the administration, the catheter was immediately removed. The newborn remained continuously on NIPPV or CPAP delivered throughout nasal mask or prongs during the procedure. All operators were skilled in performing the LISA procedure.

Dexmedetomidine administration was started 30 minutes before the LISA procedure, at a dose of 1 µg/kg intravenously over 10 minutes according to the guidelines published in the Neofarm-SIN App by the Italian Society of Neonatology [16]. Subsequently, we waited 20 minutes after the end of dexmedetomidine infusion to ensure that the drug was completely delivered after catheter wash-out and that the newborns were clinically stable. The LISA procedure was started exactly 30 minutes after the initiation of dexmedetomidine infusion to maintain consistent timing across all enrolled patients and minimize procedural variability.

In each infant, non-pharmacological comfort techniques were applied, including the administration of 24% oral sucrose in the cheek pouch/tongue with a pacifier for at least 2 minutes before the procedure, and swaddling the infant to keep them contained.

In our protocol, we did not administer atropine as it could be a confounding factor masking bradycardic events attributable to dexmedetomidine.

All infants received Caffeine (bolus 20 mg/kg intravenously) as soon as the IV line was positioned.

Infants who were excluded from the study’s protocol were managed at the discretion of the attending neonatologist. The physicians typically chose agents with which our unit has more extensive experience (typically ketamine or fentanyl).

## Data collection

We collected the following population characteristics: sex, gestational age, birth weight, Apgar score, type of respiratory support before LISA, fraction of inspired oxygen before LISA and hours of life at the time of the procedure.

Pain during the procedure was assessed using the Neonatal Infant Pain Scale (NIPS) [17] which was evaluated before, during, and after LISA procedure. This score considers various parameters (such as crying, facial expression, and arm position) and assigns a score as follows: 0–2 points = no pain; 3–4 points = mild-moderate pain; > 4 points = severe pain. We defined adequate pain control as NIPS score < 4, as we tolerated a mild pain level during laryngoscopy that is the most painful phase of LISA procedure.

Sedation was assessed using the Neonatal Pain, Agitation, and Sedation Scale (N-PASS) at the beginning of the procedure, and again at 30–60 minutes and 120 minutes. This scale evaluates both behavioral parameters (e.g., facial expression) and vital signs to assign a score, as follows: deep sedation: −10 to −6; light sedation: −5 to −2 [18]. We defined as adequate sedation a light sedation level (N-PASS score −5 to −2) in order to maintain the integrity of respiratory drive that is crucial to perform LISA.

The scores assessment was performed collaboratively by the neonatologist performing the LISA procedure and the attending bedside nurse at the time points of the protocol. All the involved staff was trained in the application of these scoring systems.

We could not fully assess the N-PASS score in four patients. In these cases, clinicians and nurses were unable to assess the sedation score because of routine clinical activities and emergency situations during their shifts.

As this study aimed to evaluate a standardized clinical protocol, the concept of blinding was not applicable.

After LISA had been performed, we collected data about the success of LISA procedure: need for intubation in the first 72 hours after the procedure, the frequencies of apnea/desaturation ±bradycardia with or without intubation in the first 72 hours after the procedure, the number of laryngoscopy attempts, the quality of intubation condition at laryngoscopy. To define the quality of intubation we used the Goldberg score, that is used in adult population, but it is easily adaptable for pediatric and neonatal population. This score considers the ease of laryngoscopy (good, fair, difficult, poor), the state of vocal cords (fully open, open, movement, closed), intubation response (none, diaphragmatic movements, moderate coughing, severe coughing). The Goldberg score defines four conditions for intubation: excellent- score < 3-, good- score 4–6-, poor score 7–9-, or inadequate- score 10–12. We defined adequate intubation condition a Goldberg score < 6, that included excellent to good intubation conditions [19].

All the operators were trained to assign NIPS, N-PASS and Goldberg scores.

Moreover, we assessed the time taken to perform LISA (defined as the time from the first laryngoscopy to the withdrawal of the catheter from the mouth) and the trend of cardiorespiratory parameters from baseline to 1, 3, 5, 15, 30, 60, and 120 minutes after the first drug injection.

Other adverse events were also collected, including pneumothorax or selective surfactant administration, as well as the incidence of adverse effects related to dexmedetomidine administration (typically cardiovascular events such as persistent bradycardia less then 100 bpm and hypotension defined as blood pressure < tenth centile for age [20].

All cardiorespiratory parameters were assessed real time by the monitors; other adverse events were collected immediately in the study electronic sheet.

Outcomes were assessed at discharge, including mortality, bronchopulmonary dysplasia (defined as the need for oxygen administration and/or respiratory support at 36 weeks postmenstrual age), intraventricular hemorrhage, necrotizing enterocolitis, and retinopathy of prematurity.

## Statistical analysis

Descriptive statistics were reported as absolute numbers (percentages) for categorical variables, and as median (I quartile – III quartile) for continuous variables. Wilcoxon’s rank sum test was used for continuous variables and the Pearson Chi-square test, or the Fisher’s exact test when appropriate (*n* < 5 in > 20% cells) were used for categorical variables to compare patient characteristics and LISA procedure-related variables between infants with gestational age 26–31^+6^ and gestational age 32–36^+6^. The NIPS and Golberg scores were dichotomized using thresholds of 4 and 6, respectively; for both scales, scores less than or equal to the cutoff indicating good clinical condition. The proportion of patients classified as being in good condition according to each scale was calculated, with 95% confidence intervals estimated using the exact binomial method.

A Linear Mixed Model (LLM) was used to assess changes in N-PASS score, saturation, and heart rate over time. LLMs extends the standard linear model by incorporating both fixed effects (parameters associated with the entire population) and random effects (parameters associated with individual experimental units or groups). This makes LMMs particularly useful for analyzing such longitudinal data, where the score was assessed at 4 different timelines (beginning − 30 – 60 - 120 minutes).

This model is also useful for missing data pattern (consistent with long term assessment) as it does not require filling in missing values and estimate the joint distribution of outcomes using available information.

A two-sided p-value of < 0.05 was considered significant. To account for multiple testing, a Benjamini-Hochberg False Discovery Rate (FDR) correction was applied across “Efficacy and safety of dexmedetomidine premedication” analyses separately [21]. An FDR-corrected p-value (termed “q-value”) threshold of < 0.05 was used to define statistical significance. All analyses were performed using R system version 4.3.2 [22].

## Ethics

The Ethics Committee of Ospedale Ca’ Foncello, Treviso, Italy, approved this observational study (number 920/CE MARCA). No additional consent was required, as the implementation of the protocol was considered standard care. All adverse events were collected.

## Results

### Population

Between May 2021 and July 2024, 82 patients were treated with surfactant for RDS. Four of them were not eligible for the LISA technique because they were aged less than 26 weeks. Seventy eight patients were eligible for our study and 31 were excluded: 7 patients were intubated in the delivery room, 4 patients with hypercapnia were intubated within the first hour of life, 2 unstable patients with hypotension and hypoxia were intubated within the first hour of life, 5 patients with hypotension only were treated with LISA and ketamine premedication, 13 patients treated with LISA and premedicated with other sedation (neonatologist choice): 4 with non-pharmacological techniques only, 4 with fentanyl and 5 with ketamine.

**Fig. 1:**
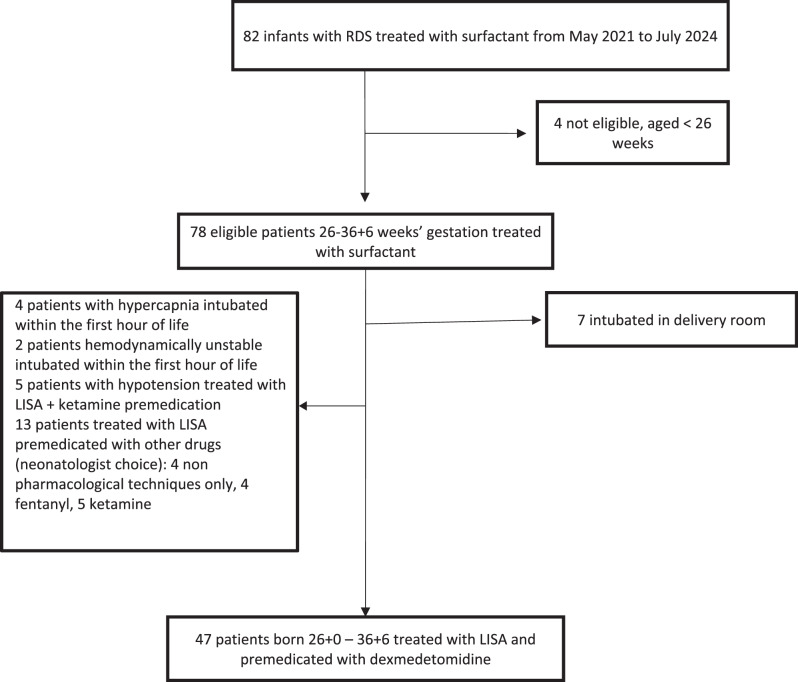
Flow-chart of the study population

Forty-seven patients were managed according to the protocol and received premedication with dexmedetomidine before the procedure (Fig. 1).

The characteristics of the population are described in Table 1, overall and divided in the two groups of age.

**Table 1:** Study population characteristics

	Overall **N = 47**^**1**^	**26–31**^**+6**^ weeks gestation **N = 30**^**1**^	**32–36**^**+6**^ weeks gestation **N = 17**^**1**^	**p-value**^**2**^
Gestational age at birth (weeks)	29+6 (28+5, 33 + 1)	29+2 (28+2, 29 + 5)	33+6 (33+0–34+5)	< 0.001
Birth weight (grams)	1421 (1069, 2074)	1130 (1005, 1362)	2180 (2046, 2340)	< 0.001
Sex Male Female	26 (55%) 21 (45%)	15(50%) 15(50%)	11 (65%) 6 (35%)	0.3
Mode of Delivery C-section n (%) Vaginal delivery	36 (77%) 11 (23%)	23 (77%) 7 (23%)	13 (76%) 4 (24%)	> 0.9
Apgar score at 5 minutes	9.00 (8.00, 9.00)	8.00 (8.00, 9.00)	9.00 (8.00, 10.00)	0.085
FiO_2_ before LISA procedure	0.35 (0.30, 0.45)	0.35 (0.30, 0.50)	0.35 (0.30–0.44)	0.6
Ventilation mode before LISA procedure nCPAP NIPPV	11 (23%) 36 (77%)	4 (13%) 26 (87%)	7 (41%) 10 (59%)	0.069
Age at LISA procedure (hours)	3.00 (2.50, 6.00)	2.80 (2.00, 4.00)	7.50 (4.00, 15)	<0.001

^1^ N(%); Median (Q1, Q3)

^2^ Fisher’s exact test; Pearson’s Chi-squared test; Wilcoxon rank sum test

Abbreviations: FiO_2_ = Fraction of inspired O_2_, nCPAP = nasal Continuous Positive Airways Pression, NIPPV: Non-invasive positive pressure ventilation, LISA = Less Invasive Surfactant Administration

Before LISA, 11 patients were treated with nasal CPAP, and 36 patients received non-invasive ventilation support, both delivered through nasal masks or prongs. The median FiO_2_ before the LISA procedure was 0.35 (I-III quartiles: 0.30–0.45), and the median time (hours) to perform LISA was 3 hours (I-III quartiles: 2.5–6). Patients born at 26–31^+6^ weeks’ gestation received surfactant at a median time of 2.8 hours (I-III quartiles: 2–4).

## Efficacy and safety of analgesia and sedation

The procedure related outcomes and the adverse events are described in Table 2, comparing them for the two age subcategories.

**Table 2:** Procedure related outcomes and premedication adverse events

	Overall N = 47	**26–31**^**+6**^ **weeks’ gestation** **N = 30**^**1**^	**32–36**^**+6**^ Weeks’ gestation N = 17	**p-value**^**3,4**^
Laryngoscopy attempts^2^	1.00 (1.00, 2.00)	1.00 (1.00, 2.00)	1.00 (1.00, 2.00)	0.6
Goldberg score (Intubation conditions)^2^ Excellent intubation conditions (score < 3) Good intubation conditions (score 4–6) Poor intubation condition (score 7–9) Inadequate intubation condition (score 10–12)	5.00 (3.00, 6.00) 12 (25,5%) 30 (63,8%) 5 (10,7%) 0 (0%)	4.00 (3.00, 5.00)	6.00 (4.00, 6.00)	0.088
LISA procedure duration (minutes)^2^	3.00 (2.00, 5.00)	3.00 (2.00, 4.00)	3.00 (2.00, 5.00)	0.6
Intubation after LISA (72 hours)^1^ Yes No	6 (13%) 41 (87%)	5 (17%) 25 (83%)	1 (6%) 16 (94%)	0.8
Time from LISA to intubation (hours) (only for patients with Intubation after LISA)^2^	2.00 (2.00, 16.00)	2.00 (2.00, 16.00)	2.00 (2.00, 2.00)	> 0.9
Apnea^1^ Yes No	6 (13%) 41 (87%)	6 (20%) 24 (80%)	0 (0%) 17 (100%)	0.6
Hypotension^1^ Yes No	3 (6.4%) 44 (93.6%)	2 (6.7%) 28 (93.3%)	1 (5.9%) 16 (94.1%)	> 0.9
Excessive sedation (N-PASS score −6 to −10)^1^ Yes No	3 (6.4%) 44 (93.6%)	2 (6.7%) 28 (93.3%)	1 (5.9%) 16 (94.1%)	> 0.9

^1^ N (%); ^2^ Median (Q1, Q3)

^3^ Fisher’s exact test; Pearson’s Chi-squared test; Wilcoxon rank sum test

^4^ False discovery rate correction for multiple testing

Abbreviations: LISA = Less Invasive Surfactant Administration, N-PASS = Neonatal Pain, Agitation, and Sedation Scale

The median of Goldberg scale value at intubation was 5 (I-III quartiles: 3–6). The 90% of newborns had scores < 6 classified as adequate intubation conditions. The median number of intubation attempts was 1 (I-III quartiles: 1–1).

**Fig. 2:**
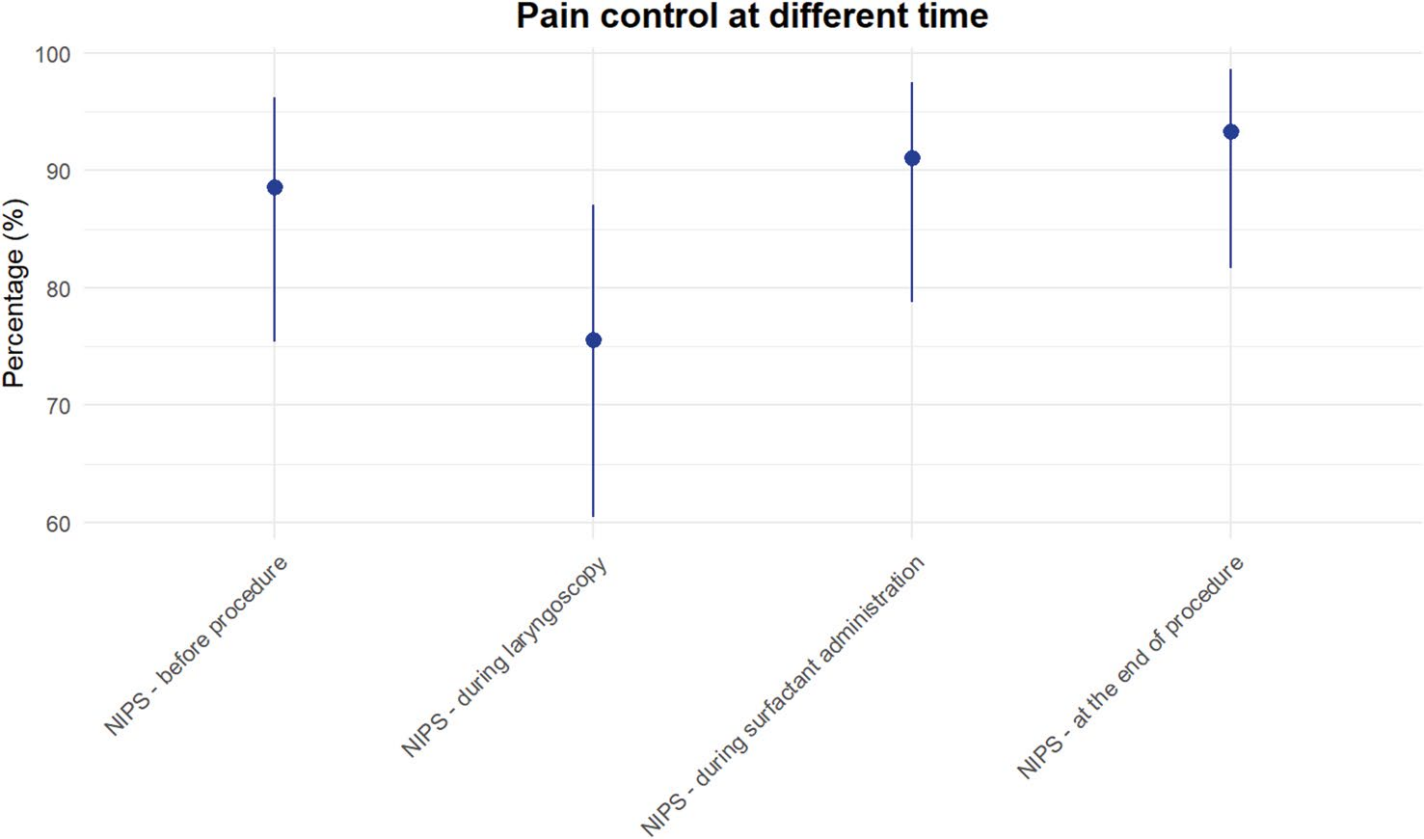
Proportion of infants with adequate pain control (NIPS score ≤4) at different phases of the LISA procedure. Points represent the estimated percentage of infants classified as being in good clinical condition, with 95% confidence intervals calculated using the exact binomial method

The NIPS pain score (median, I-III quartiles) was assessed before the procedure (1.5, 1–3), during laryngoscopy (3, 2–3), and surfactant administration (2, 1–3), as well as after the procedure (1, 0–3). Pain scores at these evaluated times showed no pain before and after the procedure, mild pain during the procedure. Before the procedure 89% of newborns had NIPS score < 4 and 76% during laryngoscopy, indicating an adequate pain control even during the most invasive part of the procedure (Fig. 2).

Sedation was assessed in 43 patients out of 47. It was evaluated using the N-PASS score (median, I-III quartiles) at the beginning of the procedure (1, 1–2), 30 minutes after the procedure (0, −2–2), 60 minutes after the procedure (0, −2–2.5), and 120 minutes after the procedure (0, 0–1.5). An adequate sedation level (N-PASS −2 to −5) was achieved in 10/43 newborns (23%) before the procedure lasting till 60 minutes after the procedure. Three patients (7%) were deeply sedated before the procedure (N-PASS −6 to −10) and this effect lasted till 120 minutes after the procedure.

**Fig. 3:**
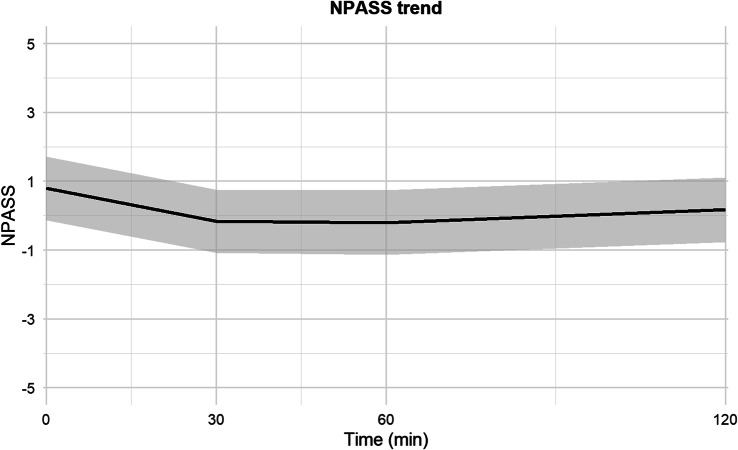
Predictive model-based trends of N-PASS score. The x-axis indicates time in minutes after the start of the LISA procedure. The y-axis shows the estimated values over time predicted by mixed linear models for N-PASS score. The continuous black line illustrates the model-predicted mean value of each variable over time. The grey area represents the 95% confidence interval for the predicted values (N-PASS= Neonatal pain, Agitation, and sedation Scale, LISA= less invasive surfactant administration)

The evolution of N-PASS distribution over time is shown in Fig. 3 as a linear predictive model.

Six patients (13%) presented apnea and desaturation/bradycardia in the first 24 hours after the procedure, of them 3 were intubated as described above. The other 3 patients were assisted successfully with NIPPV.

Six patients (13%) requested intubation within the first 72 hours after the procedure. Of these, four patients were intubated in the first 2 hours after the procedure: three for recurrent apnea/desaturation ± bradycardia (all aged less than 32 weeks) and one for a worsening of RDS (33+0). Two other patients were intubated later: one aged 30^+ 6^ weeks’ gestation at 16 hours for pulmonary hypertension and one aged 28 ^+ 2^ weeks’ gestation at 36 hours for a worsening of RDS.

Three patients developed hypotension (6.4%) in the first hours after the procedure: one extremely preterm baby (less then 28 weeks), one born from C-section after maternal blood losses and one with pulmonary hypertension. In the first two cases no pharmacological therapy was needed.

No pneumothorax or selective surfactant administration was reported.

**Fig. 4:**
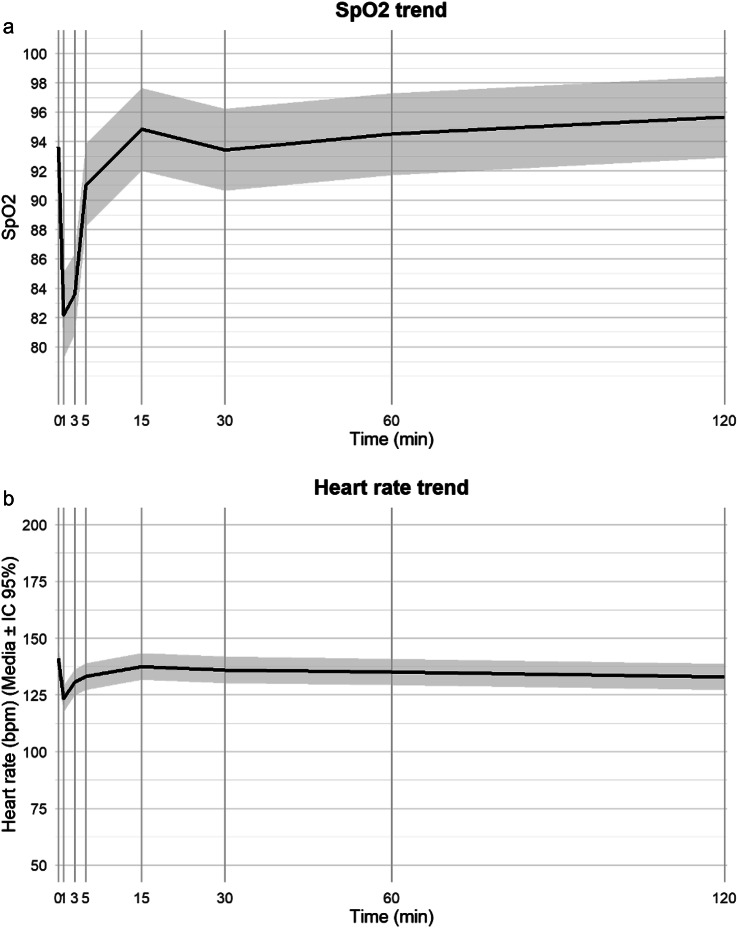
Predictive model-based trends of peripheral oxygen saturation (**A**) and heart rate (**B**) over time. The x-axis indicates time in minutes after the start of the LISA procedure. The y-axis shows the estimated values over time predicted by mixed linear models of oxygen saturation (**A**) and heart rate (**B**) the continuous black line illustrates the model-predicted mean value of each variable over time. The grey area represents the 95% confidence interval for the predicted values (SpO_2_ = peripheral oxygen saturation, LISA= less invasive surfactant administration)

Regarding vital signs in the first two hours after the procedure, the predictive evolution of oxygen saturation and heart rate from baseline is described in Figs. 4 Aand 4 B. As expected, desaturation and bradycardia increased during the maneuver from minute 1 to 5 compared to baseline, related to the obstruction to the airways while laryngoscopy was performed and surfactant administered.

## Outcome at discharge

Fourteen patients (30%) were diagnosed with BPD (13 aged between 26 and 31^+6^ weeks’ gestation). Three patients (6.4%) had minor grade IVH (less than 2), 3 patients (6.4%) had medical NEC and 11 patients (23%) had late onset sepsis. There were no deaths, no PVL. The median length of stay was 51 days (30–62).

## Discussion

To the best of our knowledge, this is the first observational prospective study describing the use of dexmedetomidine for analgesia and sedation for LISA procedure. This study was not powered to be a comparative trial and the lack of a control group should be considered in the interpretation of our findings.

We found that the administration of dexmedetomidine was associated with an adequate analgesia during laryngoscopy and good intubation conditions (evaluated by the Goldberg score).

However, light sedation was achieved in only 23% of patients during the procedure. Sedation levels closer to light sedation were observed mainly after the procedure, lasting up to 60–120 minutes. Notably, excessive sedation (N-PASS −6 to −10) occurred in 7% of patients (3 cases), with 2 of these infants extremely preterm. This suggests a higher sensitivity to dexmedetomidine in this subgroup, but no significant differences were found.

We chose dexmedetomidine to perform LISA procedure for its specific characteristics. It is a selective alpha-2 adrenergic receptor agonist that provides both sedative and analgesic effects with minimal respiratory depression [11–13, 23]. Moreover, it is a promising drug as it may offer neuroprotective benefits as demonstrated in preclinical studies [12, 13]. Continuous intravenous route/infusion has already been used in mechanically ventilated newborns, post-surgical patients, and those undergoing hypothermia treatment [24–27]. For procedural sedation, intranasal and intravenous administration has been used safely for performing MRI scans [28–30].

While a premedication for non-urgent direct laryngoscopy is strongly recommended [31, 32], there is no consensus on sedation for LISA procedure.

Surveys revealed that neonatologists in Europe [33] and in the USA [34] don’t use sedation for LISA, but they apply non-pharmacological measures such as intra-oral sucrose and swaddling. These measures seem to be effective in achieving comfort in almost 60% of preterm newborns [35].

Various drugs, including Propofol, Fentanyl, and Ketamine, have been evaluated over time, however, the supporting evidence is limited, stemming primarily from small observational, retrospective studies and only small RCTs [36–41].

Two recent systematic reviews concluded that the use of sedative drugs for LISA in preterm neonates increases the risk of desaturation and the need for NIPPV, although better comfort and pain scores (such as Comfort-Neo Score, Faceless Acute Neonatal Pain scale -FANS-) have been observed [42, 43].

Recently, a single-center study was published that tested dexmedetomidine for the LISA procedure [44]. This retrospective study by Nissimov et al. aimed to describe the incidence of adverse events (such as bradycardia or hypotension, desaturations, hypothermia) and the rate of LISA success (defined as the absence of mechanical ventilation in the 72 hours following LISA or abortion of the LISA procedure due to complications). It concluded that this drug is not associated with severe adverse events and that the rate of LISA success was favorable; however, sedation and pain control were not assessed.

A recent systematic review by Lim et al. analyzing the use of dexmedetomidine for analgesia and sedation during mechanical ventilation, suggested that it may be effective on sedation but robust data on its direct analgesic effects are lacking [45].

In our study, 76% of patients achieved analgesia even during the most painful phase of LISA—laryngoscopy—which we consider acceptable to preserve respiratory drive.

In our protocol, dexmedetomidine was not used as a sole agent for analgesia but was administered in combination with non-pharmacological interventions (such as swaddling and oral 24% sucrose).

Pichler et al. [35] demonstrated that non-pharmacological interventions are essential for achieving adequate sedation and analgesia during LISA in neonates. In their cohort, sedation was achieved in 60% of infants using these techniques alone. Consistent with these observations, our experience suggests that the application of such interventions substantially enhances pain control when combined with a low-dose pharmacological regimen.

LISA procedure is typically performed within the first hours of life in newborns with RDS, requiring an ideal drug with a rapid onset and offset of both pain control and sedation.

We infused dexmedetomidine 30 minutes before the procedure to standardize the protocol and this time seemed to be not enough to achieve an adequate sedation for an invasive maneuver.

Our findings suggest that dexmedetomidine may not be the optimal agent for this rapid procedure due to its delayed and mild sedative effect.

The low sedation rate observed likely reflects the unique pharmacokinetics of dexmedetomidine in preterm neonates. Reduced hepatic metabolism and an immature blood–brain barrier prolongs drug elimination and increase central sensitivity, while a larger volume of distribution may necessitate a loading dose [23, 46]. Longer waiting times or higher doses might improve sedation efficacy.

However, delaying the LISA procedure could compromise the optimal timing of surfactant administration, whereas administering higher doses may increase the risk of adverse events.

Regarding the safety profile of dexmedetomidine in our population, analyzing the evolution of vital signs, desaturation and bradycardia were observed in the first minutes of the LISA maneuver. These events were likely related to the procedure itself rather than to the drug administration. As Herting et al. highlighted [47], desaturation and bradycardia are commonly observed during LISA procedure, as direct laryngoscopy causes hypoxemia and bradycardia; moreover, CPAP is poorly transmitted during LISA [48, 49]. In addition, surfactant instillation itself might have negative consequences on hemodynamics as it causes transient airway obstruction [48]. These effects could potentially be mitigated by careful laryngoscopy, atropine premedication, and slow administration of surfactant. In our protocol, we did not use atropine as it could mask dexmedetomidine cardiovascular effects, but our findings suggest considering its use to prevent bradycardia during laryngoscopy.

Within the first 72 hours after the procedure, six patients required intubation (13%), 5 of them aged less than 32 weeks’ gestation, with no statistical differences between the two subcategories. Four patients were intubated within the first 2 hours after the procedure. Recurrent apnea and desaturation were noted in 3 newborns aged less than 32 weeks, which may be related to the immaturity/prematurity; however, we cannot exclude that this may be due to dexmedetomidine effects. In the other patient, intubated in the first hours, we observed a worsening of RDS. The last two patients were intubated respectively at 16 and 36 hours of life for pulmonary hypertension and a worsening of RDS. There is no direct correlation between dexmedetomidine infusion and pulmonary hypertension described in pediatric patients [50] and so we assume that in the last three cases, intubation was due to a failure of the LISA procedure itself.

Six patients presented apnea/desaturation and bradycardia (13%), all aged less than 32 weeks. Three of them were intubated and the other three were assisted with NIPPV.

Considering other cardiovascular effects of dexmedetomidine described in literature [51] hypotension was observed in three patients (6.4%). In two cases, it occurred within the first hours after administration and was managed with supportive therapy (IV fluids). One of these two newborns was born for maternal blood losses, so hypotension may not be related to dexmedetomidine infusion but to fluid depletion. The third case developed hypotension at 16 hours of life in the context of evolving pulmonary hypertension and required inotropes therapy. We did not report persistent bradycardia in the first 24 hours after the procedure.

Our findings on safety align with the retrospective study by Nissimov et al. [44], which reported a LISA success rate of 89.2% and no serious adverse events. They noted an incidence of 27% of apnea/desaturation ± bradycardia in the first 24 hours, that is a higher than ours (13%). Regarding intubation rates we noted in 13% of patients and this was similar to the Israeli report (10.8%).

The reported rates of apnea/desaturation with other tested drugs for LISA are higher. A prospective observational trial testing ketamine and atropine reported desaturations in 52% of newborns [40]. In an RCT comparing propofol with no premedication, the incidence of desaturations was 90% and 70%, respectively [38]. Another RCT comparing fentanyl with no sedation reported more than 40% of desaturations in the fentanyl group [41].

Our findings suggest that dexmedetomidine affects respiratory drive to a lesser extent. Moreover, as in Israeli court, we supported patients with NIPPV, which may also be effective in desaturations control [44].

Our results are consistent with recent reviews indicating that the major side effects of dexmedetomidine are cardiovascular, such as bradycardia and hypotension. These effects, as reported in other studies, were generally self-limiting and did not require therapeutic intervention, but only the administration of fluids or supportive care, beyond the withdrawal of the drug itself [11–13].

We observed no significant differences between the two analyzed subgroups, but we noticed that more premature infants showed a tendency to present with more adverse events such as intubations, apneas, or excessive sedation.

In their experience, Nissimov et al. used a lower median IV bolus (0.60±0.26 µg/kg) [44]. However, a retrospective analysis describing the use of dexmedetomidine for procedural sedation in newborns found a median IV bolus of 1.39 µg/kg to be safe [30]. We think that dose reduction should be considered in this vulnerable population, particularly in extremely low birth weight infants (ELBWI); however, while a lower dose might enhance the safety profile, it is unlikely to address the fundamental limitation we identified—the slow and variable onset of sedation.

Nissimov et al. [44] reported an incidence of 16.2% of mild hypothermia, suggesting that dexmedetomidine may affect thermoregulation. This parameter was not detected in our study, however the daily controls of temperature reported by the nurses did not disclosure problems.

Premedication for the LISA procedure remains a subject of ongoing debate.

To our knowledge, different drugs have been tested in retrospective and observational studies, including ketamine, fentanyl, and propofol [36–40]. Only one RCT has been conducted by a Danish group comparing propofol and no sedation, and one comparing the use of fentanyl with no sedation in a low-income country [38, 41]. All this trials together with recent systematic reviews encompassing more than 30 studies conclude that sedation reduces discomfort and pain but increases the risk of desaturation/apnea and the failure to maintain spontaneous breathing [42, 43].

These reviews underscore the need for a more standardized approach to sedation during LISA, as significant variability exists across centers in the use of both pharmacological and non-pharmacological techniques [7, 52–56].

Our experience, describing the use of dexmedetomidine as premedication for LISA procedure, support the well-known respiratory-sparing profile of dexmedetomidine, as evidenced by the lower rates of apnea and desaturation compared to published data on propofol, fentanyl, and ketamine.

Future studies should explore intranasal dexmedetomidine for LISA. The feasibility has been demonstrated in preterm infants undergoing MRI at a dose of 3 µg/kg. With an onset time of approximately 30 minutes, this route could provide more predictable sedation for LISA [28, 29].

A multimodal pharmacological approach also warrants investigation. Combining low-dose intravenous dexmedetomidine with a rapid-onset, short-acting agent such as low-dose fentanyl that has been shown to have less impact on respiratory drive compared to other agents. [38, 40, 41].

This strategy aligns with current trends in neonatal anesthesia, which favor synergistic drug combinations to minimize individual dosages and side effects.

Further research on dexmedetomidine in preterm infants should also consider lower starting doses (0.5–0.75 µg/kg), given their reduced clearance and prolonged elimination time [23].

At present, no single agent has been identified that perfectly balances the ideal characteristics of rapid onset, effective sedation and analgesia, and minimal respiratory depression for LISA procedure. Moreover, the use of atropine as premedication for LISA procedure has to be clarified.

Several promising RCT trials about premedication for LISA procedure are now ongoing: one compares propofol versus placebo [57], one the use of ketamine versus fentanyl [58], one ketamine versus placebo [59], and fentanyl along with atropine versus placebo [60].

These studies will provide further information about the best strategy for premedication before the LISA procedure.

## Limitations

This study has several limitations that must be acknowledged. The primary limitation is the prospective, single-arm, observational design, which lacks a concurrent control group. A robust comparison with our unit’s prior practice was not feasible due to considerable variability in the choice of the premedication and the absence of standardized, systematic data collection on procedural comfort and safety before the implementation of this protocol. Additionally, the open-label design introduces potential observer bias.

There are also inherent limitations to the assessment tools used. Evaluating pain and sedation using behavioral scales such as NIPS and N-PASS can be challenging, particularly during invasive procedures. Specifically, the assessment of facial expression, a key component of these scores, may be difficult during direct laryngoscopy, due to the physical containment of the infant and the presence of the laryngoscope. However, other key indicators, such as brow bulge, remain observable. Consequently, there is a potential risk of underestimating discomfort during the procedure.

To mitigate this risk, objective physiological parameters (e.g., heart rate, oxygen saturation) were collect directly from electronic monitors and subjective assessments (NIPS, N-PASS, Goldberg scores) were performed jointly by two trained clinicians to enhance inter-rater reliability.

Finally, the relatively small sample size limits the generalizability of the findings and may reduce the ability to detect rare adverse events. Moreover, the exclusion of infants born before 26 weeks’ gestation prevents direct extrapolation of the results to this particularly vulnerable population.

## Conclusion

Intravenous dexmedetomidine appears to be a safe option for premedication during the LISA procedure, as it is associated with a lower frequency of adverse events compared to other agents reported in the literature. It provides effective pain control and favorable intubation conditions. However, adequate sedation levels were not consistently achieved during the procedure and were not sustained over time.

Therefore, dexmedetomidine may not represent the optimal premedication for LISA, as a drug with a more rapid onset of action may be preferable. While awaiting more conclusive evidence from ongoing clinical trials, an individualized approach—integrating both pharmacological and non-pharmacological strategies—should be considered.

## Data Availability

Data are available from the authors upon reasonable request

## Abbreviations

RDS: Respiratory Distress Syndrome
LISA: Less Invasive Surfactant Administration
CPAP: Continuous Positive Airway Pressure
FiO_2_: Fraction of Inspired Oxygen
PEEP: Positive end-expiratory pressure
NIPPV: Non-Invasive Positive Pressure Ventilation
SpO_2_: Peripheral oxygen saturation
NICU: Neonatal Intensive Care Unit
NPASS: Neonatal Pain, Agitation, and Sedation Scale
LISA: Less Invasive Surfactant Administration
LISA: Less Invasive Surfactant Administration
NIPS: Neonatal Infant Pain Scale.
